# Can anthophilous hover flies (Diptera: Syrphidae) discriminate neonicotinoid insecticides in sucrose solution?

**DOI:** 10.1371/journal.pone.0234820

**Published:** 2020-06-19

**Authors:** C. Scott Clem, Taylor M. Sparbanie, Alec B. Luro, Alexandra N. Harmon-Threatt

**Affiliations:** 1 Department of Entomology, University of Illinois at Urbana-Champaign, Urbana, Illinois, United States of America; 2 Environmental & Plant Biology Department, Ohio University, Athens, Ohio, United States of America; 3 Department of Evolution, Ecology, and Behavior, University of Illinois at Urbana-Champaign, Urbana, Illinois, United States of America; Universitat Leipzig, GERMANY

## Abstract

Understanding how neonicotinoid insecticides affect non-target arthropods, especially pollinators, is an area of high priority and popular debate. Few studies have considered how pollinators interact and detect neonicotinoids, and almost none have examined for these effects in anthophilous Diptera such as hover flies (Syrphidae). We investigated behavioral responses of two species of hover flies, *Eristalis arbustorum* L. (Eristalinae) and *Toxomerus marginatus* Say (Syrphinae), when given a choice between artificial flowers with uncontaminated sucrose solution and neonicotinoid-contaminated (clothianidin) sucrose solution at field-realistic levels 2.5 ppb (average) and 150 ppb (high). We examined for 1) evidence that wild-caught flies could detect the insecticide gustatorily by analyzing amount of time spent feeding on floral treatments, and 2) whether flies could discriminate floral treatments visually by comparing visitation rates, spectral reflectance differences, and hover fly photoreceptor sensitivities. We did not find evidence that either species fed more or less on either of the treatment solutions. Furthermore, *T*. *marginatus* did not appear to visit one of the flower choices over the other. *Eristalis arbustorum*, however, visited uncontaminated flowers more often than contaminated flowers. Spectral differences between the flower treatments overlap with *Eristalis* photoreceptor sensitivities, opening the possibility that *E*. *arbustorum* could discriminate sucrose-clothianidin solution visually. The relevance of our findings in field settings are uncertain but they do highlight the importance of visual cues in lab-based choice experiments involving insecticides. We strongly encourage further research in this area and the consideration of both behavioral responses and sensory mechanisms when determining insecticidal impacts on beneficial arthropods.

## Introduction

Much concern has arisen about excessive use of pesticides and the resulting environmental impacts. Neonicotinoids including clothianidin, imidacloprid, and thiamethoxam are currently the most commonly used insecticides world-wide due to their low toxicity to vertebrates, high toxicity to insects, and flexibility of use [1, 2]. These synthetic compounds are applied widely in agricultural and residential settings as sprays, soil drenches, granules, and seed coatings [3, 2] where target and non-target arthropods are subject to lethal and sub-lethal effects that vary depending on dose, environmental conditions, and treatment methods [4, 5]. While the intensity of these effects is heavily contested, the consensus is that flagrant use of neonicotinoids has negative effects on non-target invertebrates and can result in changes of ecosystem structure and function [6, 5]. For example, neonicotinoids are considered one of the main contributing factors to pollinator declines that threaten numerous agricultural industries [5, 7, 8]. Most knowledge of these neonicotinoid-mediated impacts, however, are from a toxicological perspective with much less attention given to how pollinators and other non-target arthropods interact behaviorally with these insecticides.

Previous studies suggest that honey bees (*Apis mellifera* Linnaeus 1758 [9]) and bumble bees (*Bombus terrestris* (Linnaeus 1758 [9])) detect and even prefer neonicotinoid-contaminated sucrose solutions [10, 11]. Very few studies, however, have investigated for these effects on other pollinators like hover flies (Diptera: Syrphidae) which may exhibit a differential response. Honey bees and bumble bees, whose neonicotinoid-induced impacts have received the most attention relative to other pollinators, are eusocial, central-place foragers that are commonly exposed to insecticides in pollen and nectar [12]. Hover flies, on the other hand, are solitary, non-central-place foragers with life stages that occupy vastly different niches and therefore are likely be exposed to insecticides through novel means [13, 14]. Most larvae of the subfamily Eristalinae, for example, are decomposers while most Syrphinae are predators of other arthropods [15, 16]. Syrphids also possess unique physiological mechanisms which may make them pre-adapted for detecting neonicotinoids.

Flies use tarsal, antennal, wing, and labellar taste receptors for finding and detecting resources in their environment [17, 18]. Sensory hairs in the mouthparts of the hover fly *Eristalis tenax* (Linnaeus 1758 [9]), for example, have 4 to 5 receptor cells that allow them to detect differences in floral food rewards; this can influence feeding response to preferred or non-preferred foods [18]. Hover flies also rely heavily upon vision when making floral choices. Among insects, they have exceptional eyesight via a unique achromatic superposition subsystem and a tetrachromatic color vision subsystem that allows them to see a wide range of wavelengths from ultraviolet to human-visible [19–22], and this has resulted in remarkable coevolution between flies and floral traits of angiosperms [23, 24]. Recent evidence suggests that hover flies exhibit continuous color discrimination, rather than discrete color categorization, meaning that they can detect slight differences in color [25]. It is therefore possible that changes in flower spectral reflectance caused by neonicotinoid application [26, 27] may influence hover fly floral decision-making.

Here we examined the behavioral responses of two species of hover flies—*Eristalis arbustorum* (Linnaeus 1758 [9]) and *Toxomerus marginatus* (Say 1823 [28])—in a choice experiment between uncontaminated and clothianidin-contaminated (neonicotinoid) sucrose solution at average (2.5 ppb) and high (150 ppb) field-realistic concentrations. In doing so, we first explored whether flies would alter feeding behavior in response to the insecticide, possibly due to taste reception, by examining the time spent feeding on artificial flowers with uncontaminated sucrose solution compared to flowers with sucrose-clothianidin solutions. We then examined whether the hover flies would preferably visit one of the two food sources so that we could understand if the flies discriminated neonicotinoids using other sensory routes. Finally, we measured and compared hover fly-visible light reflectance on contaminated and uncontaminated artificial flower treatments. Hover flies are one of the most common and well-known groups of anthophilous Diptera that contribute significantly to crop pollination and provide other ecosystem services [29–31]. Thus, knowing how they respond to extremely common pesticide contaminants has implications for natural and agricultural ecosystems.

## Methods

### Study species and pre-trial setup

Two hover fly species common in the Midwestern United States were tested in this experiment: *Eristalis arbustorum* (Linnaeus 1758 [9]) and *Toxomerus marginatus* (Say 1823 [28]). These two species were selected because they represent the two major subfamilies of Syrphidae: Eristalinae (*E*. *arbustorum*) and Syrphinae (*T*. *marginatus*) [32] which have dissimilar life-history traits. *Eristalis arbustorum* have filter-feeding rat-tailed maggot larvae that typically live in stagnant water while *T*. *marginatus* have terrestrial, aphidophagous larvae. *Eristalis arbustorum* adults are moderately sized (10–11 mm) while *T*. *marginatus* are small (5–6 mm) [33, 34].

For these experiments, female specimens of both fly species were collected from two local prairies in Urbana, Illinois via hand-netting between the hours of 0700 and 1000 from late June to early August 2018. Flies were immediately transported to the lab and briefly chilled with ice to temporarily immobilize them. An identification color was applied on the thorax of each individual with acrylic non-toxic paint (Royal & Langnickel^®^) and flies were then placed into a 39 x 38 x 38 cm experimental arena to acclimate for two hours. The floor of each experimental arena was lined with green construction paper, three vertical walls were composed of cheese cloth, the fourth wall was a cloth sheath used for moving materials into and out of the arena, and the top of the enclosure consisted of plexiglass for viewing. During the acclimation period, arenas were provisioned with flowers that flies were found commonly feeding upon in the field: *Plantago major* (Linnaeus 1753 [35]) flowers for *T*. *marginatus*, and *Daucus carota* (Linnaeus 1753 [35]) flowers for *E*. *arbustorum*. The purpose of this was to induce feeding behavior in the flies, which preliminary trials deemed necessary. Room temperature was kept at a constant 21.5 °C and a light was installed above the arena, containing four fluorescent 120 cm bulbs: two Ecolux wide spectrum F40PL/AQ-ECO (40 W, 1,900 lm), and two Phillips Econ-o-watt F34T12/CW/RS/EW (34 W, 2,650 lm). After the two-hour acclimation period, real flowers were replaced with experimental, artificial flowers and the trial was commenced.

### Feeding trial

Flies were offered two choices of artificial flowers per experimental trial: a flower with clothianidin-laced sucrose solution, and a control flower with uncontaminated sucrose solution. Flowers were plastic with 8 cm diameter white petals and a 1 cm yellow central disk (Hobby Lobby^®^ product #662601), resembling a generic, white aster flies would encounter in the field. Efforts were made to ensure test flowers were equal in size and shape before each trial. The two test flowers were placed in 50 ml flasks, each arranged in the middle half of the arena, opposite of one another. Labels designating treatment and control were placed in front of each flower, and the order of the two flowers was randomized before each trial. Webcams (Logitech^®^ 1080p) were placed on the plexiglass and set to record fly behavior for six hours per trial.

Three *E*. *arbustorum* or ten *T*. *marginatus* females were used in each experimental trial; these numbers were chosen based on ease of capture in the field. A total of 40 trials were conducted, split into four types of trials at 10 trials per species per clothianidin concentration. Two different concentrations of clothianidin (Sigma-Aldrich PESTANAL^®^, product #33589) in 0.5 M sucrose solution were tested: 2.5 ppb and 150 ppb. Clothianidin at 2.5 ppb is an average field-realistic level in oilseed rape nectar [36] while 150 ppb is an upper-end lawn level [37]. 200 μL of the chosen solution was pipetted onto the central disk of each artificial flower. At the end of each trial, artificial flowers were disposed and replaced with new flowers to begin the next trial.

Data were collected by examining videos and recording visitation and feeding behaviors. Each time a fly visited a flower, the flower identity (clothianidin or sucrose), fly identity (color), and amount of time spent feeding per each visit was recorded. A fly was considered feeding if its proboscis was extending and retracting over the sucrose in a feeding motion, and a visit was defined as each time a fly landed on a flower and fed.

### Light measurements

A JAZ spectrometer (Ocean Optics, Inc.) with a pulsed-xenon light source and 400 μm bifurcated fiber optic cable fitted with a reflectance probe for 90° angle measurements was used to record reflectance spectra from the centers of artificial flowers with 1) freshly applied clothianidin-sucrose solution at 150 ppb and dried for one hour after application and 2) freshly applied sucrose solution alone and dried one hour after application. We also measured the reflectance of pure clothianidin powder (Sigma-Aldrich PESTANAL^®^, product #33589) against black aluminum foil (ThorLabs Inc., product #BKF12; spectral reflectance ≤ 5% from 300–700 nm). We examined freshly applied vs. dried solutions to examine potential differences in reflectance spectra caused by the drying process. Reflectance measurements from each flower treatment (10 total: sucrose + clothianidin freshly treated or dried centers and petals, sucrose-only freshly treated or dried centers and petals, and dry untreated centers and petals) were taken in triplicate relative to a WS-1-SL 99% white reflectance standard (Labsphere).

### Statistical analyses

#### Bayesian multilevel models

We used the *brms* package [38], an extension of the *rstan* package [39] in R 3.6.1 [40] to estimate differences in hover fly feeding response times and number of visits to each flower treatment. For each hover fly species, we ran two Bayesian models to test hover fly responses to flower treatments: model 1) estimating hover fly total feeding response times, and model 2) estimating hover fly total count of flower visits. Both models were used to examine i) *E*. *arbustorum* response to the 2.5 ppb clothianidin vs. sucrose-only treated flowers, ii) *E*. *arbustorum* response to 150 ppb clothianidin vs. sucrose-only treated flowers, iii) *T*. *marginatus* response to the 2.5 ppb clothianidin vs. sucrose-only treated flowers, and iv) *T*. *marginatus* response to the 150 ppb clothianidin vs. sucrose-only. Response variation between experimental trials for each species (i.e., experimental trial as a “random effect”) was accounted for in all applications of the models. Models were run for 10,000 iterations for 4 chains to obtain an effective sample size > 2000 for each model parameter.

#### Model priors

The model estimating time spent feeding (model 1) by hover flies used a Gaussian response distribution with a log_e_ link function. Weakly informative prior distributions were included for the population mean feeding response time (i.e., intercept) (Cauchy: location = 6, scale = 2), the standard deviation of population mean feeding response time (Student T: df = 3, location = 0, scale = 10), predictor effects of two-choice experiment type (sucrose only vs. sucrose + 2.5 ppb clothianidin, or sucrose only vs. sucrose + 150 ppb clothianidin), hover fly species (*E*. *arbustorum* or *T*. *marginatus*), the artificial flower which was fed on (sucrose only or sucrose + clothianidin), and all of the predictors’ interactions (Student T: df = 10, location = 0, scale = 2). We also included a prior for the model’s residual standard deviation (Student T: df = 3, location = 0, scale = 10).

The model estimating the total number of flower visits by hover flies (model 2) used a Poisson response distribution with log_e_ link function. Weakly informative prior distributions were included for the population mean number of flower visits (i.e., intercept) (Cauchy: location = 0, scale = 0.6), the standard deviation of the population mean number of flower visits (Student T: df = 3, location = 0, scale = 2), and the same predictors and predictor interactions as model 1 (Student T: df = 30, location = 0, scale = 1.5).

#### Differences in hover flies’ responses between treatments and hypothesis tests

We estimated pairwise differences in hover flies’ feeding and visitation responses to the flower treatments (i.e., feeding/visit response towards the sucrose only or sucrose + clothianidin-treated flower) by taking the differences between posterior distributions of hover fly responses towards each flower treatment under each trial condition (sucrose only vs. sucrose + 2.5 ppb clothianidin, or sucrose only vs. sucrose + 150 ppb clothianidin) using the *emmeans* package in R [41]. Additional pairwise differences in hover flies’ responses were also estimated across trial conditions to test if hover flies differed in their responses towards specific flower treatments under different trial conditions (e.g., did hover flies feed more on the sucrose-only flowers in the trials where the alternative choice was a sucrose + 2.5 ppb clothianidin-treated flower or a sucrose + 150 ppb clothianidin-treated flower?), if overall feeding/visitation responses to all flowers differed between trial conditions (e.g., did hover flies feed more during sucrose only vs. sucrose + 2.5 ppb clothianidin trials or sucrose only vs. sucrose + 150 ppb trials?), and if hover flies differed in their responses to sucrose only vs. both clothianidin-treated flowers across all trials (i.e., did hover flies feed more on sucrose-only flowers than sucrose + clothianidin treated flowers?). We then performed Region of Practical Equivalence (ROPE) tests [42] using the *bayestestR* package in R 3.5.1 [40] to assess both if hover flies discriminated or did not discriminate (null) between artificial flower treatments and the magnitude of discrimination between artificial flower treatments. We set our null ROPE interval threshold as response differences greater than 10% of the standard deviation of the mean response [42]. A useful aspect of ROPE null hypothesis tests is that, unlike frequentist null hypothesis tests, existence of a predictor’s effect on a response can be tested as either likely, null, or undecided (i.e., insufficient evidence in either direction to be determined) [42].

ROPE null hypothesis tests were done by calculating the percent of the 95% highest posterior density interval (HPDI) of the response difference (all responses on log_e_ scale) that lies within the ROPE interval, setting <2.5% overlap of the 95% HDPI within the ROPE interval as our null hypothesis rejection threshold [42]. We set a ROPE interval of -0.15, +0.15 for both hover fly feeding time and flower visitation responses (±1.16 seconds feeding/flower visits). The ROPE overlap rejection threshold is inherently subjective, and we chose <2.5% as our threshold to follow convention of α = 0.05 used in frequentist hypothesis testing (also a subjective value). ROPE overlap percentage values roughly correspond to frequentist *p*-values and are a metric for testing the existence and magnitude of an effect [43]. For example, a “rejected” null hypothesis equivalence test for an estimated difference in feeding response time between flower treatments from model 1 where the ROPE overlap value is 1% would indicate a feeding response time difference between treatments greater than ±1.16 seconds (i.e., ±0.15 log_e_ seconds–Table 1) with a reasonable degree of confidence. The ROPE null hypothesis test would be “accepted” if the entire response 95% HDPI is within the ROPE region (ROPE overlap = 100%) and we would conclude that hover flies lack discrimination between artificial flower treatments when feeding. An “undecided” null hypothesis ROPE test (ROPE overlap >2.5% and <100%) should be interpreted as insufficient evidence to support either a complete lack of discrimination by hover flies between experimental flower treatments (evidence in favor of null) or the existence of hover fly discrimination between flower treatments (evidence against null).

**Table 1:** Hover fly feeding response time differences (log_e_) between two-choice experimental treatments and across all experimental treatments.

Comparison	Species	Treatment Comparison	log_e_ Feeding Time Median ± MAD	95% Highest Density Interval	ROPE Overlap	Equivalence Test
Pairwise						
	*Eristalis arbustorum*					
		sucrose only 2.5 ppb—sucrose + CLO 2.5 ppb	0.08 ± 0.35	-0.63, 0.87	36%	Undecided
		sucrose only 2.5 ppb—sucrose only 150 ppb	-0.12 ± 0.34	-0.81, 0.61	34%	Undecided
		sucrose + CLO 2.5 ppb—sucrose + CLO 150 ppb	0.19 ± 0.63	-1.18, 2.03	21%	Undecided
		sucrose only 150 ppb—sucrose + CLO 150 ppb	0.39 ± 0.59	-0.82, 2.27	19%	Undecided
	*Toxomerus marginatus*					
		sucrose only 2.5 ppb—sucrose + CLO 2.5 ppb	0.43 ± 1.27	-2.32, 3.89	10%	Undecided
		sucrose only 2.5 ppb—sucrose only 150 ppb	0.81 ± 1.38	-1.85, 4.42	8%	Undecided
		sucrose + CLO 2.5 ppb—sucrose + CLO 150 ppb	1.54 ± 2.18	-2.55, 6.77	5%	Undecided
		sucrose only 150 ppb—sucrose + CLO 150 ppb	1.2 ± 2.14	-2.91, 6.42	6%	Undecided
All Experiments						
	*Eristalis arbustorum*					
		sucrose only—sucrose + CLO	0.25 ± 0.36	-0.51, 1.27	30%	Undecided
	*Toxomerus marginatus*					
		sucrose only—sucrose + CLO	0.86 ± 1.51	-2.12, 4.62	8%	Undecided

“Pairwise” comparison is sucrose only vs. sucrose + CLO (clothianidin), 2.5 ppb or 150 ppb. “All experiments” comparison: sucrose only vs. sucrose + CLO flower, 2.5 ppb and 150 ppb combined. Region of Practical Equivalence (ROPE) null hypothesis tests were done against the null ROPE interval of (-0.15, 0.15; or 1.16 seconds difference in hover fly feeding response), where smaller ROPE overlap percentages indicate greater confidence in the presence of a difference in response, and ROPE overlaps < 2.5% are the null hypothesis rejection threshold.

### Ethics statement

Experimental flies were collected on University of Illinois at Urbana-Champaign property with the prior permission of the Committee on Natural Areas. To minimize suffering, flies were immediately euthanized after each experiment by placing them in a freezer.

## Results

### Hover fly response models

**Fig 1:**
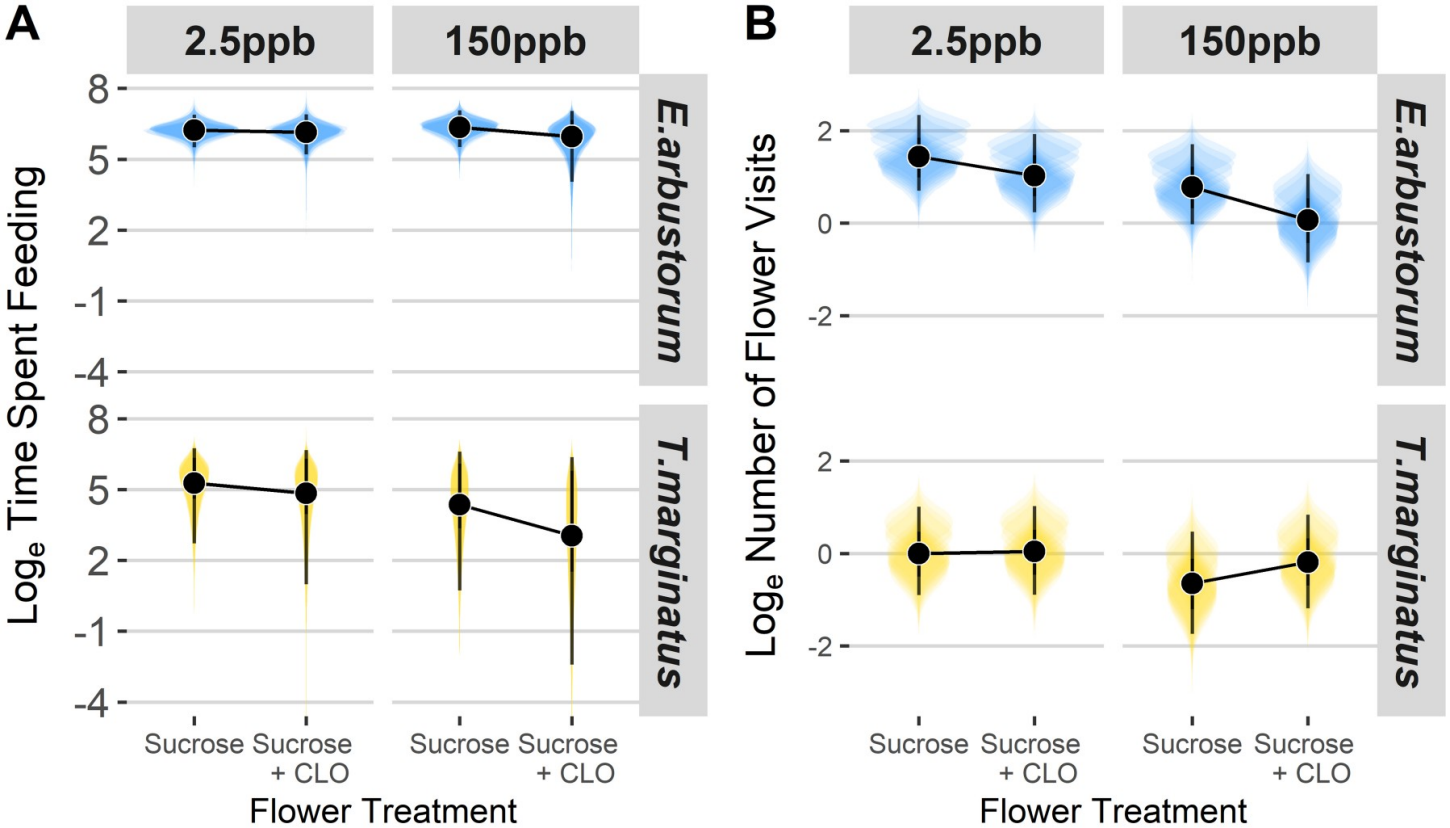
Hover fly time spent feeding and number of visits to sucrose only vs. sucrose + clothianidian (CLO) treated artificial flowers. Median and 95% highest posterior density intervals of (A) log_e_ time spent feeding on sucrose only vs. sucrose + clothianidin (CLO) treated artificial flowers at 2.5 ppb and 150 ppb and (B) log_e_ number of visits to sucrose only vs. sucrose + clothianidian (CLO) treated artificial flowers at 2.5 ppb and 150 ppb for *Eristalis arbustorum* and *Toxomerus marginatus* hover flies. Shaded areas respresent posterior distributions of hover flies’ responses for individual trials (n = 10 trials).

All model results were analyzed separately for each hover fly species, *Eristalis arbustorum* and *Toxomerus marginatus*. Markov Chain Monte Carlo iterations reached convergence for both models (Rhat = 1.0 for all model estimates) [44], and all model estimate posterior draw effective sample sizes were > 7000. Medians and 95% highest posterior density intervals of time spent feeding and number of flower visits in response to each treatment for both species are presented in Fig 1 and S1 Table.

### Hover fly feeding time responses to flowers

Region of Practical Equivalence (ROPE) null hypothesis tests were done against the null ROPE interval of (-0.15, 0.15; or ±1.16 seconds difference in hover fly feeding response). There was insufficient evidence to determine if *E*. *arbustorum* or *T*. *marginatus* fed significantly more or less than ±1.16 seconds (i.e., ±0.15 log_e_ seconds–Table 1) between any two flower treatments (all ROPE overlaps > 2.5% and < 100%) (Table 1, Fig 1A).

### Hover fly visitation response to flowers

Region of Practical Equivalence (ROPE) null hypothesis tests were done against the null ROPE interval of (-0.15, 0.15; or ±1.16 visits difference in hover fly flower visitation response). Models testing differences in visits found evidence that *E*. *arbustorum* visited flowers treated with sucrose alone more often than sucrose + clothianidin flowers in trials with 150 ppb clothianidin solution, by a difference of 2 ± 1.4 visits (posterior median ± median absolute difference, percent overlap with region of practical equivalence: 0.72 ± 0.34 log_e_ difference in flower visits, 2% ROPE overlap) (Table 2, Fig 1B). *Eristalis arbustorum* also visited artificial flowers treated with sucrose alone by about 1.76 ± 1.23 visits more often than flowers treated with sucrose + clothianidin across both 2.5 ppb and 150 ppb treatment two-choice experiments (0.57 ± 0.21 log_e_ difference in flower visits, 0% ROPE overlap), but it was undetermined whether *T*. *marginatus* had any preference for visiting either flower treatment (-0.25 ± 0.33 log_e_ difference in flower visits, 28% ROPE overlap). Finally, we also found that *E*. *arbustorum* visited flowers of the 2.5 ppb clothianidin treatment two-choice experiment more often than flowers of the 150 ppb two-choice experiment when comparing both visits between sucrose alone treated flowers (0.67 ± 0.24 log_e_ difference in flower visits, 0% ROPE overlap) and sucrose + clothianidin-treated flowers (0.97 ± 0.32 log_e_ difference in flower visits, 0% ROPE overlap) (Table 2). Flower visitation differences were unclear for *T*. *marginatus* across all treatment comparisons (all ROPE overlaps > 2.5% and < 100%).

**Table 2:** Hover fly flower visit response differences (log_e_) between two-choice experimental treatments and across all experimental treatments.

Comparison	Species	Treatment Comparison	log_e_ Flower Visits Median ± MAD	95% Highest Density Interval	ROPE Overlap	Equivalence Test
Pairwise						
	*Eristalis arbustorum*					
		sucrose only 2.5 ppb—sucrose + CLO 2.5 ppb	0.42 ± 0.22	-0.01, 0.86	9%	Undecided
		sucrose only 2.5 ppb—sucrose only 150 ppb	0.67 ± 0.24	0.24, 1.16	0%	Rejected
		sucrose + CLO 2.5 ppb—sucrose + CLO 150 ppb	0.97 ± 0.32	0.35, 1.62	0%	Rejected
		sucrose only 150 ppb—sucrose + CLO 150 ppb	0.72 ± 0.34	0.08, 1.4	2%	Rejected
	*Toxomerus marginatus*					
		sucrose only 2.5 ppb—sucrose + CLO 2.5 ppb	-0.05 ± 0.39	-0.81, 0.7	31%	Undecided
		sucrose only 2.5 ppb—sucrose only 150 ppb	0.64 ± 0.46	-0.24, 1.58	10%	Undecided
		sucrose + CLO 2.5 ppb—sucrose + CLO 150 ppb	0.24 ± 0.43	-0.58, 1.09	25%	Undecided
		sucrose only 150 ppb—sucrose + CLO 150 ppb	-0.45 ± 0.5	-1.48, 0.49	17%	Undecided
All Experiments						
	*Eristalis arbustorum*					
		sucrose only—sucrose + CLO	0.57 ± 0.21	0.17, 0.99	0%	Rejected
	*Toxomerus marginatus*					
		sucrose only—sucrose + CLO	-0.25 ± 0.33	-0.91, 0.4	28%	Undecided

“Pairwise” comparison is sucrose only vs. sucrose + CLO (clothianidin), 2.5 ppb or 150 ppb. “All experiments” comparison: sucrose only vs. sucrose + CLO flower, 2.5 ppb and 150 ppb combined. Region of Practical Equivalence (ROPE) null hypothesis tests were done against the null ROPE interval of (-0.15, 0.15; or 1.16 seconds difference in hover fly feeding response), where smaller ROPE overlap percentages indicate greater confidence in the presence of a difference in response, and ROPE overlaps < 2.5% are the null hypothesis rejection threshold.

### Light measurements

**Fig 2:**
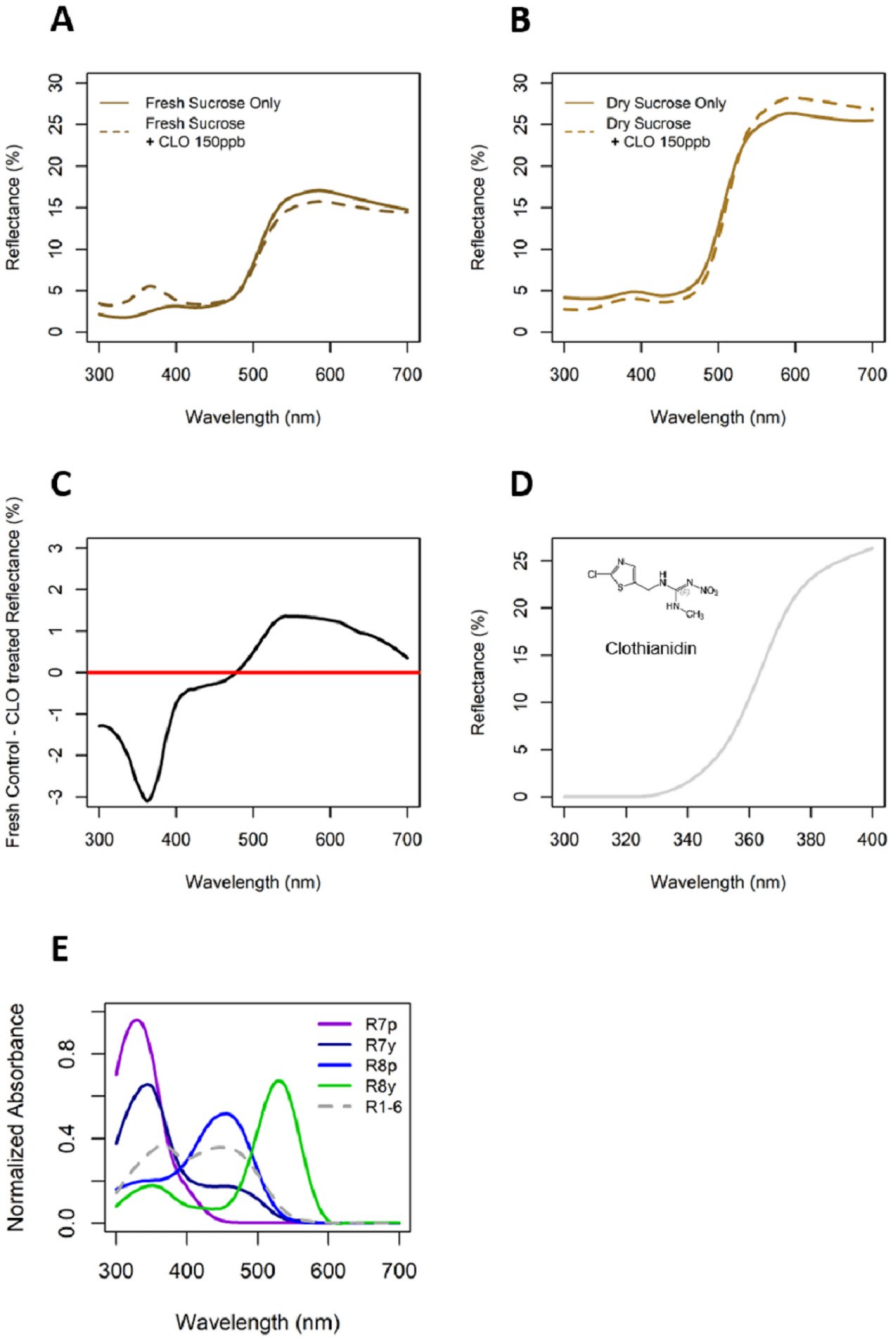
Hover fly-visible reflectance spectral measurements (300–700 nm). (A) freshly treated and (B) dried flower centers for sucrose only (solid) and sucrose + 150 ppb clothianidin (dashed line) treatments. (C) Difference in reflectance spectra of fresh sucrose only (control) vs. sucrose + 150 ppb clothianidin. (D) Ultraviolet reflectance spectra (300–400 nm) of pure clothianidin powder against black optical aluminum foil (< 3% reflectance across all 300–700 nm). (E) *Eristalis tenax* photoreceptor sensitivities as normalized absorbance curves [19, 45].

Reflectance spectra measurements reveal that freshly applied sucrose + 150 ppb clothianidin solution on artificial flower disks exhibited a slight increase in the UV range around 360–400 nm in comparison to artificial flowers disks with freshly applied sucrose only solution (Fig 2A and 2C). Similar to the freshly applied clothianidin-laced sucrose solution, we found that pure dry solid clothianidin powder reflects UV light around 360–400 nm (Fig 2D), suggesting that clothianidin in aqueous solution may increase the UV reflectance. As the solutions applied on flower disks were given an hour to dry, however, the difference in UV reflectance diminished (Fig 2B). Flower disks with freshly applied sucrose + 150 ppb clothianidin solution also had slightly lower reflectance from 500–600 nm (green/yellow for humans) (Fig 2C) than flower disks with fresh sucrose-only solution, but this difference in reflectance reversed when flower disks were dry (Fig 2B).

## Discussion

In this study, we found that some hover fly species may be capable of detecting the neonicotinoid clothianidin, and that the presence of this insecticide may influence hover fly decision making. We found inconclusive evidence whether *Toxomerus marginatus* and *Eristalis arbustorum* altered feeding behavior in response to differences in clothianidin-laced sucrose and uncontaminated sucrose. Neither species spent significantly more or less time feeding on either of the two flower treatments. Feeding time data for *T*. *marginatus*, however, did approach the ROPE overlap rejection threshold, suggesting that the species may have spent less time feeding on sucrose + clothianidin at 150 ppb (Table 1, Fig 1). This contrasts with bumble bees (*B*. *terrestris*) and honey bees (*A*. *mellifera*) which were both found to prefer neonicotinoid-laced solutions [10, 11]. Lack of a significant trend in our study may be due to an increased hover fly tolerance for neonicotinoids. Flies used in the experiment were taken from prairies surrounded by conventional agriculture; these populations could have had reduced sensitivity due to constant neonicotinoid exposure. Hover fly larvae may be directly exposed to insecticides in agricultural fields, drainage ditches, and waste lagoons which may cause adults to be less sensitive to contaminants in their food. Larvae of *T*. *marginatus* and other Syrphinae, for example, likely interact with insecticides via their aphid (Hemiptera: Aphididae) prey which are often direct targets of chemical control. *Eristalis* may be particularly pre-disposed to tolerating insecticides because their larvae can survive in extreme, anoxic environments where they break down organic waste-products [46]; indeed, larvae of *Eristalis tenax*, a congener of *E*. *arbustorum*, was shown to tolerate high doses of thiamethoxam, only exhibiting lethal and sublethal effects at 500 ppb [13]. To better understand whether hover flies can discriminate neonicotinoids in their food sources via taste, however, electrophysiology should be used to examine taste-receptor stimulation.

Despite inconclusive evidence that clothianidin affects time spent feeding by either *E*. *arbustorum* or *T*. *marginatus*, it did appear that *E*. *arbustorum* could detect presence of this neonicotinoid on artificial flowers. Individuals of this species visited artificial flowers with sucrose alone more frequently than flowers with clothianidin-laced sucrose, especially at high doses (Table 2, Fig 1). These results align with that of Easton and Goulson (2013)[47], who found that pan traps laced with imidacloprid in a field setting collected significantly fewer flies and beetles compared to pan traps without the insecticide. It is unlikely that the flies in our experiment could detect the insecticide through olfaction as clothianidin is not considered highly volatile [48, 49], so we explored the possibility that it could be detected visually. Our light reflectance measurements reveal that both fresh sucrose + 150 ppb clothianidin solution on artificial flowers and pure clothianidin powder reflects ultraviolet (UV) light (Fig 2A, 2C and 2D). *Eristalis arbustorum* likely have R7p and R7y photoreceptors with sensitivity to light in the UV range (Fig 2E), so they may be attuned to differences in UV reflectance and either 1) prefer UV-deficient flower centers, and/or 2) avoid flowers with high UV reflectance in their centers. This has precedence in the literature, as the presence of UV light is known to inhibit hover fly proboscis extension [22, 50]. Naïve *E*. *tenax* flies were also reported to prefer UV-absorbing over UV-reflecting yellow colored disks [19]. In our experiments, however, we used fluorescent lighting which is not known to emit significant amounts UV light, and any potential UV reflectance likely dissipated within an hour from the start of an experimental trial, so it is uncertain if flies could distinguish this. The two freshly-applied flower treatments also differed in the 500–600 nm wavelength range (Fig 2A–2C), near the predicted peak wavelength sensitivity of the hover fly R8y photoreceptor (Fig 2E), opening the possibility that differences between freshly applied sucrose only vs. sucrose + 150 ppb clothianidin treated flower disks in reflectance of this wavelength range was more apparent to the flies throughout the experiment (Fig 2A and 2B). Evidence that *Eristalis* can detect differences this precise, however, have yet be demonstrated [25], so we do not believe our findings to be definitive evidence that these flies could visually detect the neonicotinoid treatment. *Toxomerus marginatus* did not appear to exhibit differences in visitation between any of the floral treatments. We do not know whether this species could have been sensitive to these flower disk reflectance differences because their photoreceptor wavelength sensitivities are currently unknown.

Assertions as to how neonicotinoid spectral changes affect hover flies in the field should be approached with caution. If hover flies can detect neonicotinoids under field-relevant light sources, we would predict that UV reflectance caused by neonicotinoids may become weaker over time (Fig 2A and 2B), so this would likely limit the salience of UV reflectance of neonicotinoids in the field. We would also predict that the rate and route of neonicotinoid application is likely to influence neonicotinoid perceivability. In cases where plants uptake neonicotinoids systemically, visual detectability would depend on the structure of floral nectaries, exposure of nectar, and the prevalence of the insecticide. Factors like the presence of UV-absorbing sucrose in a solution are also expected to alter light reflectance [51]. Perhaps these compounds would be most impactful in situations where flowers and vegetation have been directly sprayed with neonicotinoids at high concentrations, or in cases where hemipteran honeydew found on vegetation surfaces is contaminated with high concentrations of neonicotinoids. Indeed, imidacloprid and thiamethoxam can occur in honeydew at significant-enough levels (upwards to 98 ppb imidacloprid and 290 ppb thiamethoxam per honeydew volume) under standard applications, which can affect hover flies and parasitoids [52]. Finally, hover flies make decisions based on both olfactory and visual cues [50], and in some cases olfactory cues can be more important [53]. Therefore, slight spectral differences caused by neonicotinoids may be less significant in a field scenario than olfactory cues like floral scent [53]. We strongly suggest that rigorously controlled, field-based studies are necessary to truly test hover flies’ capabilities of visually detecting neonicotinoids.

Our examination highlights the importance of insect visual sensitivities in insecticide-based choice experiments, which may partially explain why pollinators preferred or avoided neonicotinoid-laced sucrose solutions in previous studies. The light source for these studies is important; in cases where natural sunlight is used, reflectance caused by clothianidin or other neonicotinoids may be more apparent than in our study due to a greater amount of UV light. Visual cues resulting from spectral alteration caused by insecticides are seldom, if ever, considered as major factors that may influence insect behavior. Indeed, along with hover flies, other pollinating insects including bees and butterflies possess visual sensitivity to ultraviolet light [20, 54], so it is possible that neonicotinoids are detectable by multiple taxa. We therefore stress that in order to fully understand how insects interact with insecticides, one must consider visual cues and other routes of detection.

## Conclusions

In our experiment we found inconclusive evidence that two hover fly species, *E*. *arbustorum* and *T*. *marginatus*, spent more or less time feeding on sucrose solution contaminated with the neonicotinoid clothianidin. We did, however, find that *E*. *arbustorum* visited artificial flowers laced with clothianidin-contaminated sucrose solution less often than flowers with uncontaminated sucrose solution. Based on light reflectance measurements and hover fly photoreceptor sensitivities, we believe it possible that the flies may have been capable of detecting clothianidin visually, which may partially explain the visitation data. These data highlight the importance of investigating not only the toxicity of insecticides, but also how beneficial insects perceive and interact with them in their environment. Continued over-use of neonicotinoid insecticides in landscapes world-wide makes understanding the extensiveness of neonicotinoid impacts on non-target organisms a pursuit of paramount importance.

## Supporting information

S1 Table Summary statistics for hover fly feeding and visitation data. (DOCX)
