# Supplementary material for: Can anthophilous hover flies (Diptera: Syrphidae) discriminate neonicotinoid insecticides in sucrose solution?
Source: PLoS One. 2020 Jun 19;15(6):e0234820. doi: 10.1371/journal.pone.0234820 (PMC7304583; doi:10.1371/journal.pone.0234820)
Supplement: S1 Table — (DOCX) [file pone.0234820.s001.docx]

**Table_S1. Summary statistics for hover fly feeding and visitation data.** Medians and 95% highest posterior density intervals of time spent feeding and number of flower visits in response to each treatment for *Eristalis arbustorum* and *Toxomerus marginatus*.

| Species | Flower Treatment | Dose Trial | log_e_ Time Spent Feeding  Posterior Median, 95% HPD | log_e_ Number of Flower Visits  Posterior Median, 95% HPD |
| --- | --- | --- | --- | --- |
| *Eristalis arbustorum* |  |  |  |  |
|  | Sucrose | 2.5ppb | 6.23, 5.72 - 6.66 | 1.45, 1.02 - 1.87 |
|  | Sucrose + CLO | 2.5ppb | 6.15, 5.41 - 6.73 | 1.04, 0.54 - 1.49 |
|  | Sucrose | 150ppb | 6.35, 5.7 - 6.86 | 0.78, 0.26 - 1.26 |
|  | Sucrose + CLO | 150ppb | 5.97, 4.14 - 6.92 | 0.07, -0.56 - 0.7 |
| *Toxomerus marginatus* |  |  |  |  |
|  | Sucrose | 2.5ppb | 5.29, 2.83 - 6.69 | -0.01, -0.67 - 0.59 |
|  | Sucrose + CLO | 2.5ppb | 4.86, 1 - 6.56 | 0.04, -0.62 - 0.65 |
|  | Sucrose | 150ppb | 4.38, 0.8 - 6.57 | -0.66, -1.54 - 0.12 |
|  | Sucrose + CLO | 150ppb | 3.05, -2.29 - 6.36 | -0.2, -0.95 - 0.45 |
